# Novel Sutureless Scleral Fixated IOL for Inadequate or Absent Capsular Support

**DOI:** 10.1155/2022/2161003

**Published:** 2022-01-25

**Authors:** Georgios Sidiropoulos, Elisabeth Siskou, Spyridon Koronis, Paris Tranos, Zisis Gatzioufas, Miltos Balidis

**Affiliations:** ^1^Ophthalmica Eye Institute, Thessaloniki, Greece; ^2^Department of Ophthalmology, University Hospital Basel, Basel, Switzerland

## Abstract

**Purpose:**

To evaluate the clinical outcome and safety profile of a new sutureless scleral fixation (SSF) technique using a single-piece foldable acrylic Carlevale intraocular lens.

**Methods:**

In this case study, 27 eyes of 27 patients were implanted with an SSF single-piece IOL because of inadequate or absent capsular support. The hand-shake technique used during surgery was combined with the creation of scleral pockets in order to secure the IOL haptics. The BCVA was evaluated in the 1st and 6th month in every patient and in the 12th and 24th months, when possible. Also, we evaluated the improvement achieved in spherical equivalent values from baseline to the 6th month after the procedure. Intraoperative and postoperative complications were assessed.

**Results:**

The mean age was 69.1 ± 14.9 years, and the mean follow-up was 13.6 ± 4.8 months. Indications of scleral-fixated IOL included dislocated posterior chamber IOL (40.7%), dislocated anterior chamber IOL (11.1%), subluxated traumatic cataract (18.5%), subluxated nontraumatic cataract (18.5%), and aphakia (11.1%). Concurrent PPV was performed on eight of the eyes (32%). The mean preoperative logMAR BCVA increased from 0.85 ± 0.59 baseline to 0.44 ± 0.30 one month after surgery (*p* < 0.01) and 0.36 ± 0.34 (*p* < 0.003) six months after surgery. The baseline refractive status expressed in SE was 4.3 ± 6.4 D, and the postoperative status was −0.5 ± 0.99 D. Postoperative complications included vitreous hemorrhage (7.4%), hypotony (7.4%), transient IOP elevation (3.7%), and postoperative cystoid macular oedema (3.7%). The IOL was very well centered and stable in every case during the follow-up period.

**Conclusion:**

The use of the SSF technique with implantation of a single-piece foldable acrylic Carlevale IOL seems to be a safe and effective alternative method that provides good preliminary results in cases where capsular support is inadequate or absent. Long-term stability results would be required to evaluate the benefit of this novel surgical approach in order to compare it with other existing methods.

## 1. Introduction

Cataract surgery with intraocular lens (IOL) implantation is currently one of the most frequent and successful surgical procedures [1]. However, when capsular support is inadequate or absent, IOL may be challenging even for experienced surgeons. Several techniques have been employed over the years to deal with zonular dehiscence or dialysis. Among the most common of these techniques are iris fixation suturing or iris-claw [2–6], anterior chamber IOL implantation [7], scleral fixation IOL with suturing [8], and the most recent: sutureless intrascleral IOL fixation [9–12], and glued IOL.

Sutureless intrascleral fixation was initially introduced by Maggi et al. in 1997, followed by the tunnel fixation method, proposed by Gabor Scharioth, and later modified as glued transscleral fixation by Agarwal et al. [9]. Recently, Yamane proposed “flanged fixation” [8–12]. This so-called Yamane technique externalizes the haptics of a three-piece IOL using a thin-walled 30- or 27-gauge needle inserted through two transconjunctival sclerotomies. Each haptic of the IOL is carefully placed into the lumen of the needle using intraocular forceps. Then, the needle is used to externalize each haptic on the conjunctival surface, followed by low-temperature cautery to make a flange or bulb at the edge of the haptics. This flange prevents the haptics from prolapsing back into the posterior chamber. Thus, the IOL is fixated efficiently in the posterior segment in the absence of capsular support.

Moreover, scleral-fixated IOL implantation is considered to be a safe procedure, in that the results show a reduction in suture-related complications [13] and in induced astigmatism. However, when 3-piece IOLs are used for scleral fixation, their long-term stability is debatable, since such IOLs are not specifically designed for this purpose. [8–12].

**Figure 1:**
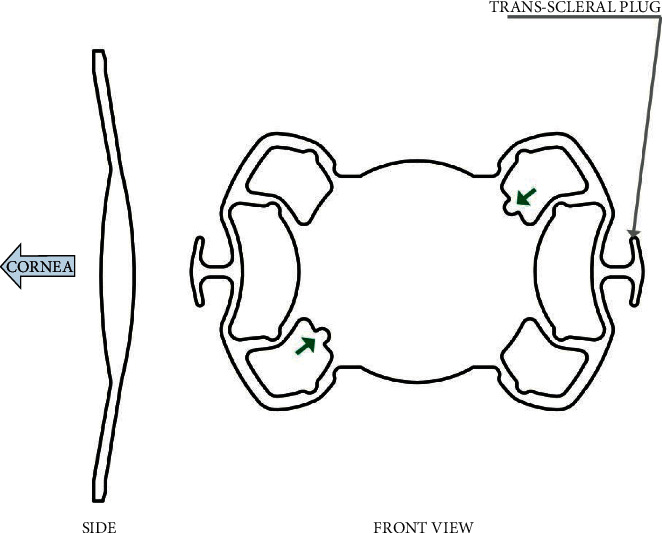
Demonstration of the single-piece SSF-IOL Carlevale Lens.

In this prospective analysis, we present a novel surgical technique for sutureless scleral-fixated IOL using a single piece SSF - IOL Carlevale Lens® (Soleko IOL Division, Italy, Figure 1). The Carlevale IOL is a foldable, one-piece, acrylic, monofocal, scleral fixating IOL that has a flexible anchorlike plug on the end of each haptic (Figure 1: demonstration of the single-piece SSF-IOL Carlevale lens). The current technique used during IOL implantation is the hand-shake technique, followed by the creation of scleral pockets in which the IOL haptics are secured afterward.

## 2. Patients and Methods

A novel surgical technique of sutureless scleral-fixated IOL implantation was performed on 27 eyes of 27 patients at the Ophthalmic Eye Institute between February 2019 and September 2020. All the surgeries were conducted by a single surgeon.

Inclusion criteria: patients had to be over 18 years old; BCVA > finger counting; patients had to have dislocated posterior or anterior chamber IOL, aphakia, and traumatic cataract with weak capsular support. Exclusion criteria: patients could not have extended glaucomatous or macular damage.

All patients provided written consent prior to surgery and the tenets of the Declaration of Helsinki were fully respected. The clinic's ethics committee approved the study, and the approved number was 01/2019/003_OPH_Aphakia. Baseline characteristics are summarized in Table 1 (baseline characteristics). The mean follow-up was 13.6 ± 4.8 months. Twenty-one patients received at least 12 months of follow-up and the other six, six months. Eleven of them went on to receive 24 months of follow-up.

The patients underwent a full preoperative examination including best corrected visual acuity (BCVA) in Snellen decimals, which were converted to the logarithm of the minimum angle of the resolution equivalents (LogMar), refraction, slit-lamp biomicroscopy of the anterior and posterior segment, Goldmann applanation tonometry (GAT IOP mmHg), fundus examination, and endothelial cell count density (Tomey EM -3000). A follow-up was scheduled on the 1st day, and also for the1st, 3rd, 6th, 12th, and 24th months.

During the follow-up, BCVA, GAT-IOP, and intra or postoperative complications were noted and evaluated. Refraction was also evaluated as spherical equivalent (SE) for the 1st and 6th months and compared to the baseline values. The IOL power was calculated using optical biometry (Lenstar, Haag-Streit). Whenever optical biometry was not possible, a standard conventional A-Scan biometry was employed to measure the axial length (AL mm), which was then fed into Lenstar to calculate the IOL power. All IOLs were calculated with the SRK-T, Barret universal, and Haigis formula.

Hypotony was defined when IOP was less or equal to 5 mmHg, while transient ocular hypertension was defined when IOP was equal to or more than 22 mmHg at any visit.

When pars plana vitrectomy (PPV) was employed (8 eyes), the data were analyzed and reported separately.

Statistical analysis was performed using MedCalc® 16.2.1 and IBM SPSS® statistics version 22. Parametrical or nonparametrical tests were used according to distribution. A *p* < 0.05 was considered statistically significant.

## 3. Surgical Technique

All surgeries were performed under retrobulbar anesthesia. Following anesthesia, corneal markings on the 10–190° axis were performed to ensure the correct centration of the IOL. A nasal and temporal conjunctival peritomy was then performed, followed by cauterization of the sclera under based saline solution (BSS) irrigation. Two spots were marked 1.5 mm behind the limbus to correspond to the corneal markings on the 10°–190° axis. A nasal and temporal sclerotomy was then performed at this location using an MVR 23 gauge knife (Alcon Grieshaber DSP Sterile Disposable) at a vertical orientation.

**Figure 2:**
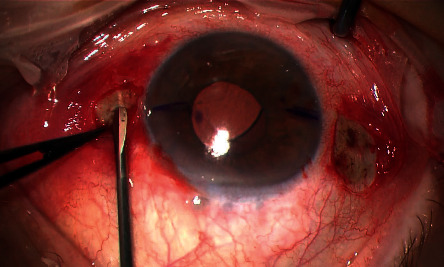
Creation of scleral pockets bilateral of the scleral tunnel.

With each sclerotomy, two lateral midscleral 1.0 mm tunnels were created by dissecting the sclera perpendicularly to the incision. In this manner, two opposite self-sealing pockets were fashioned to position the two ends of the transscleral plug. This step was a modification of the technique proposed by Veronese et al. [14] (Figure 2).

In cases of the subluxated crystalline lens, phacoemulsification was performed with Stellaris Elite™ (Bausch and Lomb, USA) via a 2.2 mm corneal one-step incision using capsule hooks to support the weak zonules. Displaced IOLs were extracted from a 2.75 mm corneal three-step incision. After the removal of the IOL or the crystalline lens, an anterior and core vitrectomy was executed.

When required, a 25G or 27G PPV was performed. A 25G PPV was executed with Stellaris Elite™ (Bausch and Lomb, USA) when there was a loss of IOL or nucleus in the vitreous cavity. When macular pathology coexisted, a 27G PPV was preferred.

**Figure 3:**
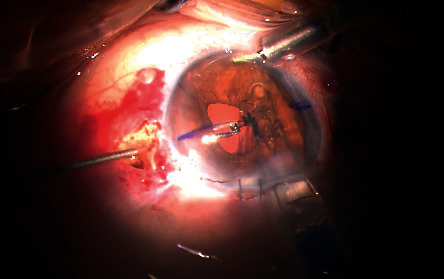
Leading plug grasped by crocodile tip forceps.

**Figure 4:**
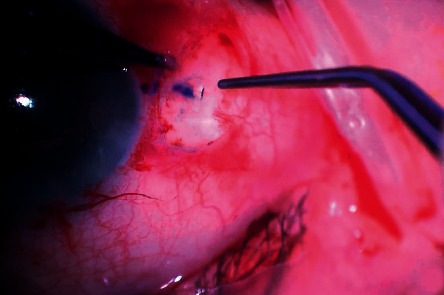
Transscleral plug placed in the scleral pocket.

A single-piece hydrophilic Carlevale IOL was inserted into the anterior chamber with the IOL injector. Microintraocular forceps were employed through the sclerotomy to grasp the plug of the IOL haptic to prevent the IOL from falling into the vitreous cavity (Figure 3). Two notches on the IOL body (one on the lower left side and one on the upper right) helped the surgeon check the IOL's orientation (Figure 1). Then, the forceps were slowly withdrawn, dragging the plug through the sclerotomy. The trailing plug was grasped and externalized with the hand-shake technique using two 25G intraocular forceps. Lastly, the two ends of each plug were positioned inside the scleral pockets that had been created for this purpose (Figure 4). The dimensions of the anchorlike “transscleral plugs” were 2 mm in width and 1 mm in length. Following the IOL centration, the sclerotomies were tested for leaks and the conjunctiva was closed, if needed, with an 8/0 absorbable polyglactin (vicryl suture). In three cases (all myopic eyes), the sclerotomies displayed leakage and the sclera was sealed with a 9/0 polypropylene suture to correct the problem.

## 4. Results

A preoperative evaluation gave evidence of weak capsular support due to trauma in six eyes (22.2%), pseudoexfoliation syndrome in two eyes (7.4%), and Marfan and Weill-Marchesani syndrome in two eyes (7.4%). In the cases, 40.7% presented posterior chamber IOL dislocation; 11.1% presented anterior chamber IOL dislocation; 18.5% had preexisting subluxated traumatic cataracts, and 18.5% displayed subluxated nontraumatic cataracts. Lastly, 11.1% of the cases presented preoperative aphakia (from a previous operation). Eight eyes (29.6%) underwent concurrent 25 or 27 G pars plana vitrectomy (PPV). The mean axial length was 22.5 ± 0.68 mm (range 21.5 to 24.2 mm) and the mean IOL power was 19.3 ± 2.97 D (range 12 to 26 D) (Table 2).

The mean BCVA increased from 0.85 ± 0.59 LogMar baseline to 0.44 ± 0.30 at one month (Wilcoxon test, *p* < 0.01) and to 0.36 ± 0.34 (Wilcoxon test, *p* < 0.003) at six months. The refractive spherical equivalent also changed significantly from 4.3 ± 6.4 D to −0.5 ± 0.99 D at six months (*p* < 0.01 paired samples *t*-test). No change in the BCVA was observed at the 12-month follow-up. The mean corneal endothelial cell density had decreased from 2472 ± 202 cells/mm^2^ to 2387 ± 197 cells/mm^2^ (paired samples *t*-test, *p* < 0.01) (Table 2.)

In the PPV group, mean BCVA increased from 1.02 ± 0.60 LogMar baseline to 0.65 ± 0.37 within the first month (Wilcoxon test, *p* < 0.01) and to 0.47 ± 0.30 (Wilcoxon test, *p* < 0.005) within six months. The refractive spherical equivalent also changed significantly from 3.6 ± 12 D to −0.59 ± 0.98 D within six months (*p* < 0.01 paired samples *t*-test). The mean corneal endothelial cell density decreased from 2553 ± 205 cells/mm^2^ to 2453 ± 200 cells/mm^2^ (paired samples *t*-test, *p* < 0.01) (Table 2).

Postoperative complications included vitreous hemorrhage in two eyes (7.4%), which resolved without intervention; hypotony in two eyes (7.4%), which resolved automatically after three days; and transient hypertony in one eye (3.7%) on the 1st day, which was treated medically (Table 3). There were no signs of uveitis. Postoperative optical coherence tomography was performed on every patient (SPECTRALIS OCT, Heidelberg Engineering, Heidelberg, Germany), revealing one case of postoperative cystoid macular oedema (CMO). During the follow-up period, there was neither haptic exposure nor scleral or conjunctival erosion. Hypotony in one eye (12.5%) was the only postoperative complication in the PPV group.

## 5. Discussion

Poor capsular support may be observed in many ocular conditions such as trauma or pseudoexfoliation syndrome, or as an ophthalmic manifestation in systemic pathologies such as Marfan and Weill-Marchesani syndrome. Many surgical procedures have been proposed over the years to address IOL support in the absence of an intact capsule. The three options surgeons have been anterior chamber IOL, iris fixated IOL, and scleral fixated IOL. The percentages of complications vary among different studies. A report by the American Academy of Ophthalmology in 2003 compared the efficacy of secondary IOLs and concluded that there is insufficient evidence to demonstrate the superiority of one lens type or fixation site [15]. Each of these methods has advantages and disadvantages that should be taken into consideration.

The new technique of sutureless scleral fixation using the single-piece foldable Carlevale IOL, which is designed specifically for this purpose, offers considerable advantages for the surgeon. Its specially designed sclero-corneal plugs prevent the reinsertion of the haptic into the vitreous cavity. Placement of the plugs in the scleral pockets offers good IOL stability [14, 16]. The 13.5 mm total length and 6.5 mm large optic improve the centration and function of the lens. The Carlevale IOL's specially-designed soft haptics can be stretched, increasing the total length for severe myopia and Marfan cases [17]. The learning curve of this method is relatively steep, meaning that extensive surgical exposure to a significant number of cases is required in order to gather the experience that is necessary for mastering this technique. One possible disadvantage is the haptic and plug fragility. Compared to the conventional 3-piece IOLs, IOL haptics can be torn with negligible force during manipulation. Therefore, it is very important for the IOL to be well-centered with minimal effort.

In this study, overall visual acuity showed a statistically significant improvement, and the refractive outcome was acceptable in all cases. Several studies evaluating alternative methods for secondary IOLs placement, such as the iris-fixated [18], sutureless scleral-fixated [19–22], and anterior chamber [23] methods, reported rates of transient ocular hypertension ranging from 4% to 12.4% [18–24], IOL dislocation in 0–12% [18–24], hyphema in 4.0–9.7% [18–20, 23], vitreous hemorrhage in 0–12.2% [18–23], serous choroidal detachment in 1.3–2.7% [18, 19, 23], IOL capture within uveal tissue in 0–8.6% [19, 21, 23], cystoid macular oedema in 0–6.9% [18–24], retinal detachment in 0–2% [19–24], and anterior uveitis in 1.1–5.4% [18] cases. In the present study, the most common complications were vitreous hemorrhage (9.5%) and transient hypotony (9.5%). Similar results were shown in the study by Barca et al. [16] Regarding the IOL stability, there were no dislocations during the examined period. The corneal endothelium remained intact and there were no noticeable signs of inflammation in the postoperative anterior chamber. Altering the classic technique minimized the risk of scleral or conjunctival erosion since no portion of the lens was exposed during the examined period.

In summary, we have reported our clinical results of using sutureless scleral fixation of the Carlevale IOL, which seems to be a safe and effective method, providing good visual outcomes in situations where capsular support is inadequate or absent. The Carlevale IOL's main advantage is its special design for scleral fixation, which offers unique characteristics, one of which is good stability with minimum need of intraoperative manipulation to achieve perfect centration. Long-term results and evaluation of outcomes are needed to determine the superiority of this procedure compared with other, more well-established ones.

One limitation of our study is the small sample size. However, we propose a longer follow-up time for this new approach to SFIOL using this novel IOL.

## Figures and Tables

**Table 1:** Baseline characteristics.

		Total	PPV group	Non PPV group
Total patient		27	8	19
Age ± SD		69.12 ± 14.9 years (range 44–91)	61.25 ± 17.5 years (range 45–91)	72.6 ± 12.5 years (range 44–90)
Gender	Male	18 (66.6%)	14	4
	Female	9 (33.3%)	5	4
Follow up		13.6 ± 4.08 months (range 26–6)	14 ± 6.2 months (range 26–6)	13.5 ± 4.4 months (range 20–6)

**Table 2:** Results.

	Total	PPV group	Non PPV group
Preoperative logMAR BCVA	0.85 ± 0.59 (range 0.05–2.3)	1.02 ± 0.60 (range 0.05–1.8)	0.83 ± 0.61 (range 0.15–2.3)
1-month postoperative logMAR BCVA	0.44 ± 0.30 (range 0.05–1)	0.65 ± 0.37 (range 0.15–1)	0.37 ± 0.35 (range 0.05–0.95)
6 months postoperative logMAR BCVA	0.36 ± 0.34 (range 0.05–1)	0.47 ± 0.30 (range 0.05–1)	0.32 ± 0.33 (range 0.05–0.95)
12 months postoperative logMAR BCVA	0.35 ± 0.32 (range 0.05–1)	0.50 ± 0.30 (range 0.05–1)	0.33 ± 0.32 (range 0.05–0.95)
24 months postoperative logMAR BCVA	0.33 ± 0.32 (range 0.05–0.9)	0.45 ± 0.27 (range 0.05–0.9)	0.30 ± 0.31 (range 0.05–0.6)
Endothelial cell count	Preoperative	2472 ± 202 cells/mm^2^	2553 ± 205 cells/mm^2^
	Postoperative	2387 ± 197 cells/mm^2^	2453 ± 200 cells/mm^2^
Indication	Dislocated PC IOL (40.7%)		
	AC IOL complication (11.1%)		
	Subluxated traumatic cataract (18.5%)		
	Subluxated nontraumatic cataract (18.5%)		
	Aphakia (11.1%)		

**Table 3:** Postoperative complications.

Postoperative complications	Eyes (%)
Vitreous hemorrhage	2 (7.4%)
Hypotony	2 (7.4%)
Hypertony	1 (3.7%)
CMO	1 (3.7%)

## Data Availability

The SPSS file which contains the data used to support the findings of this study is available from the corresponding author upon request.
